# Transcriptome and WGCNA Reveal the Key Genes of Arbuscular Mycorrhizal Fungi in Regulating Sugarcane Growth and Nutrient Absorption

**DOI:** 10.1002/fsn3.70508

**Published:** 2025-07-03

**Authors:** Lifang Mo, Ziqin Pang, Zhuowei Tan, Qiang Liu, Yixian Jia, Yijie Xiao, Zhaonian Yuan

**Affiliations:** ^1^ National Engineering Research Center for Sugarcane Fujian Agriculture and Forestry University Fuzhou China; ^2^ Xianghu Laboratory Hangzhou China; ^3^ Agricultural Products Quality and Safety Supervision and Management Station of Qintang District Guigang China; ^4^ College of Natural Resources and Environment Northwest A&F University Yangling China

**Keywords:** arbuscular mycorrhizal fungi, RNA‐seq, soil nutrients, sugarcane growth, WGCNA

## Abstract

Sugarcane is a major sugar crop with substantial economic importance worldwide. Arbuscular mycorrhizas (AM), which are capable of forming symbioses with the majority of terrestrial plants, play a pivotal role in plant productivity and soil nutrient cycling. The present study employed pot experiments using a randomized block design. Two treatments were applied: inoculation with AM fungi (A20) and non‐inoculation (CK), each with nine replicates within each block. A comparative analysis was conducted on the phenotypic traits, rhizosphere soil nutrient contents, and transcriptomics of sugarcane during the elongation stage. The results demonstrated that AM fungal inoculation not only facilitated the accumulation of above‐ and belowground biomass in sugarcane but also significantly influenced the contents of soil organic matter, available nitrogen, available phosphorus, and total phosphorus in the rhizosphere. The utilization of RNA‐seq and Weighted Gene Co‐expression Network Analysis (WGCNA) enabled the identification of genes and modules associated with sugarcane growth and nutrient absorption during the elongation stage following AM fungal inoculation. In particular, the MEmoccasin and MElightpink3 modules were identified as being highly specific to nutrient phenotypes, including nitrogen and phosphorus, as well as belowground growth. In contrast, the MEhoneydew1 module was found to be specifically associated with aboveground phenotypic traits, such as plant height and stem diameter. It is noteworthy that CESA9, ANR, CYCP4, LHA1, SUS4, RPS15AE, and CNGC2 were identified as potential hub genes, exerting crucial regulatory functions in soil carbon cycling, nitrogen and phosphorus content, and sugarcane growth. This study provides insights into the effects of AM fungi on sugarcane growth and nutrient absorption, establishing a theoretical foundation for further understanding the molecular mechanisms underlying AM fungal influence on these processes in sugarcane.

## Graphical abstract

AM fungal inoculation boosted sugarcane biomass and improved rhizosphere soil nutrient levels. RNA‐seq and WGCNA revealed gene modules linked to nutrient uptake and growth, differentiating above‐ and belowground responses. Hub genes (CESA9, ANR, CYCP4, LHA1, SUS4, RPS15AE, CNGC2) were identified as key regulators of sugarcane growth and nutrient cycling.

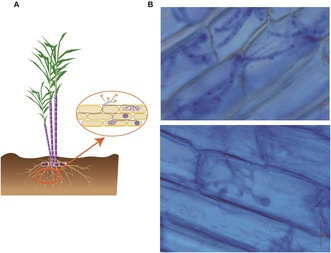

## 1 Introduction

Sugarcane, as the cornerstone crop in the global sugar industry and bioenergy sector, boasts considerable annual yields, thereby occupying a pivotal position in the global agricultural economy (Garsmeur et al. 2018; Pang, Fallah, et al. 2021). As the depth of cultivation practices intensifies and the industrial scale expands, the challenges and issues encountered in sugarcane cultivation have become increasingly apparent, posing pivotal constraints to its sustainable development. On one hand, continuous cropping in sugarcane cultivation ensures land efficiency but triggers yield decline and soil acidification, adversely affecting soil nutrients (Pang, Tayyab, et al. 2021; Tayyab et al. 2021). On the other, sugarcane's peak nutrient demand during elongation directly impacts stem growth, crucial for yield and quality. Heavy fertilizer application, the common response, leads to unsustainable nutrient loss, soil acidification, and hardening, intensifying ecosystem stress and increasing farmer costs (Wang et al. 2024). Therefore, it is crucial for the development of sustainable agriculture to enhance fertilizer use efficiency and reduce production costs while simultaneously achieving high yields in sugarcane cultivation.

Arbuscular mycorrhizal (AM) fungi represent a class of microorganisms capable of forming symbiotic mycorrhizal associations with the roots of most terrestrial plants (Pan et al. 2020; Shi et al. 2024). These fungi penetrate the plant root epidermis through hyphae, entering into the root epidermal cells where they develop into arbuscules and vesicles, structures crucial for signal exchange and nutrient transfer. Simultaneously, the fungal growth extends into the soil, developing extraradical mycelia (ERM) that primarily participates in the absorption of mineral nutrients and the production of spores (Roth and Paszkowski 2017; Zhang, Zhou, et al. 2022). Numerous studies have demonstrated that arbuscular mycorrhizal fungi can facilitate the absorption of nutrient elements such as carbon, nitrogen, phosphorus, potassium, sulfur, and iron in host plants (Bao et al. 2018; Gou et al. 2024; Martin and van der Heijden 2024; Qin et al. 2023; Shuming et al. 2021; Teranishi and Kobae 2020; Xie et al. 2022), stimulate plant growth (Kabir et al. 2024; Kou et al. 2024), and enhance crop yields (Bolin et al. 2022; Chen et al. 2018). Furthermore, they can improve crop water use efficiency (Xu et al. 2024), bolster plant disease resistance and stress tolerance (Bahadur et al. 2019; Chen et al. 2023; Dan et al. 2023; Dowarah et al. 2021), and enhance product quality, among other benefits.

In recent years, transcriptome sequencing has emerged as a pivotal technological tool in biology, with an increasing number of studies leveraging this technology to delve into data or potential mechanisms (Rohan et al. 2017). Weighted Gene Co‐expression Network Analysis (WGCNA) is a systematic biological methodology utilized to delineate patterns of gene associations among different samples. Through the construction of gene co‐expression networks, WGCNA reveals the interconnectivity between genes and clusters them into distinct modules, aiding in the identification of gene sets that exhibit similar functionalities or are regulated in similar manners (Yu et al. 2023). For example, WGCNA has been widely adopted to pinpoint hub genes implicated in diverse biological questions such as specific biological processes (Li et al. 2022; Song et al. 2021; Wang et al. 2018), responses to abiotic stresses (Azad et al. 2024; Kitavi et al. 2023; Wang et al. 2022), and pathogen resistance (Liu et al. 2021; Wu et al. 2022).

Although existing studies have demonstrated that AM fungi can significantly promote nutrient uptake and growth in various crops, the molecular mechanisms underlying these effects in sugarcane remain largely unexplored. Improving nutrient use efficiency, reducing fertilizer application, and achieving sustainable development are urgent priorities in the sugarcane industry. However, there is a substantial knowledge gap regarding which gene networks are regulated by AM fungi and how these networks specifically influence sugarcane growth and nutrient absorption at the molecular level. The central scientific question of this study is: How do AM fungi modulate gene expression networks to promote nutrient uptake and growth in sugarcane? Based on this, we hypothesize that the symbiotic relationship with AM fungi activates specific gene modules related to nutrient transport, cell expansion, and growth regulation, thereby accelerating sugarcane growth and enhancing nutrient utilization efficiency. To test this hypothesis, we employed transcriptome sequencing and WGCNA to identify key genes and module networks involved in regulating sugarcane growth and nutrient absorption. This study is innovative: by systematically applying WGCNA, it reveals the molecular regulatory networks underlying the sugarcane‐AM fungus interaction, providing molecular insights for optimizing microbial‐assisted breeding and management strategies to promote the sustainable development of the sugarcane industry.

## 2 Materials and Method

### 2.1 Experimental Site and Materials

The experiment was conducted on April 3, 2023, within the greenhouse facilities of the Sugarcane Research Base at Fujian Agriculture and Forestry University (FAFU), located at 26°4′ N, 119°14′ E in Fuzhou, China. The inoculum comprised a blend of five arbuscular mycorrhizal fungal species: *Claroideoglomus etunicatum*, *Claroideoglomus claroideum*, *Rhizophagus irregularis*, *Funneliformis geosporum*, and *Funneliformis mosseae*. The experimental setup utilized a soilless growing medium, with maize, *Tagetes erecta* (marigold), and *Trifolium repens* (clover) as host plants. The AM fungal spores were inoculated into these hosts and allowed to establish for a period of 5 months. Following this period, the spores, host root systems, and the soilless medium were collectively harvested and prepared as inoculum material for future use. Each kilogram of the AM fungal inoculum contains 500,000 AM fungal spores. The experimental sugarcane cultivar utilized in this study is ROC22, specifically sourced from the prestigious Sugarcane Germplasm Resource Nursery of Fujian Agriculture and Forestry University, ensuring the authenticity and representativeness of the soil substrate employed for our investigations.

### 2.2 Experimental Design and Sample Collection

The experimental setup consisted of two treatments: a control group and a bacterial inoculation treatment group, each with nine biological replicates, totaling 18 pots. The pots were arranged according to a randomized complete block design. Sterilized sandy soil was weighed and placed into disinfected plastic barrels capable of holding approximately 16 kg of soil mixture (about half of the barrel volume). The propagated mixed microbial inoculum (2 g, containing approximately 1000 spores) was introduced into the center of each planting barrel. Then, sugarcane seedlings were transplanted into the barrels, ensuring that the roots came into direct contact with the microbial inoculum. Finally, about 5 cm of sterilized sand and soil mixture was layered over the seedlings. The pots were kept in a plastic greenhouse with the temperature maintained between 25°C and 40°C during summer, and relative humidity controlled within 60%–80% RH. The barrels were placed on plastic sheeting following a randomized block design, with periodic adjustments to their positions. Watering was conducted every 1–2 days, with the amount depending on the moisture status and weight change of the substrate within each barrel, supplementing with evaporated water to maintain suitable moisture levels. Fertilization was based on the fertilization standards of the main sugarcane producing areas (750 kg/hm^2^), which corresponded to approximately 0.75 g per barrel of compound fertilizer with an N:P:K ratio of 15:5:15, and a total nitrogen, phosphorus, and potassium content ≥ 35%.

Sampling during the sugarcane jointing stage entailed excision of approximately 2 g of roots and stem segments, which were meticulously rinsed with sterile water, dried with blotting paper, and disinfected with 75% alcohol‐soaked cotton. These samples, replicated three times per treatment, were promptly wrapped in aluminum foil, flash‐frozen in liquid nitrogen, and stored in an ultra‐low temperature (−80°C) freezer. Concurrently, 0–20 cm soil samples were collected via the five‐point method, homogenized, purified, and sealed in sterile bags with treatment labels (*n* = 3 per treatment). The soil samples were dried in a ventilated environment for subsequent nutrient content analysis (Aurélien et al. 2022).

### 2.3 Measurement of Phenotypic Traits and Rhizosphere Soil Physico‐Chemical Indicators in Sugarcane

In rigorous scientific investigation, the height, stem diameter, and brix of experimental sugarcane were precisely measured using calibrated instruments. Soil pH was analyzed with a high‐precision meter (model PB‐10, Sartorius, Göttingen, Germany), adhering to a 2.5:1 water‐to‐dry soil ratio. SOC quantification employed the potassium dichromate oxidation method, while AN was assessed via alkaline hydrolysis. AP was extracted with sodium bicarbonate and TP measured spectrophotometrically. Soil available potassium was determined by ammonium acetate extraction and flame photometry (Kwon et al. 2009; Scrimgeour 2007; Tang et al. 2020). AM fungal hyphal colonization in sugarcane roots was evaluated following (McGonigle et al. 1990) methodology.

The plant height, stem diameter, and brix (hammer degree) of the experimental sugarcane plants were measured using a height gauge, vernier caliper, and PAL‐GrapeMust (Brix) refractometer, respectively. The photosynthetic rate and chlorophyll content were determined using a portable photosynthesis and chlorophyll fluorescence measurement system (Li‐6800) and a chlorophyll meter, respectively. Grayscale scanning of the planar images of the sugarcane root system was conducted at 300 dpi using an Epson Expression 12000XL root scanner. Subsequently, the WinRHIZO root analysis system was employed to analyze root length and root surface area.

### 2.4 RNA Extraction, Library Construction, and Sequencing

During the elongation phase, roots (R20), and stems (S20) of AM‐colonized sugarcane, alongside untreated controls (Rck, Sck), were harvested for RNA‐seq. Total RNA was extracted, evaluated for purity, concentration, and integrity, then sent to Biomarker Technologies in Beijing for analysis. There samples underwent total RNA inspection, quality assessment, cDNA library construction, and transcriptome sequencing.

The raw data were filtered using FastQC software, and the resulting clean reads were utilized for subsequent transcriptome analysis. TopHat v2.0.9 was employed to align the paired‐end clean reads to the reference genome, followed by HTSeq v0.6.1 to count the number of reads aligned to each gene on the genome (https://phytozome‐next.jgi.doe.gov/info/SofficinarumxspontaneumR570_v2_1). By normalizing the read counts, the RPKM (Reads Per Kilobase Per Million) value for each gene was calculated. Subsequently, the probability of the hypothesis test (*p*‐value) was computed based on the negative binomial distribution model. The *p*‐values were then corrected for multiple hypothesis testing using the Benjamini‐Hochberg method to obtain the FDR (False Discovery Rate). Finally, DESeq was utilized to analyze differentially expressed genes (DEGs) between two distinct samples, with DEGs selected based on a criterion of *p* value ≤ 0.05 and a fold change of at least 2 in expression levels.

### 2.5 Co‐Expression Network Construct and Analysis

Based on the expression correlation patterns among differentially expressed genes, we performed WGCNA (Langfelder and Horvath 2008). A threshold of FPKM value equal to 1 was applied to filter out lowly expressed genes. The weight values were calculated using the WGCNA analysis toolkit on the cloud platform of Biomarker Technologies Co. Ltd. (www.biocloud.net), Beijing, to ensure that the network conforms to a scale‐free network distribution. Gene clustering trees were constructed based on the correlation of expression levels between genes, and gene clustering and module partitioning were performed using a dynamic tree‐cutting algorithm, with a minimum gene count of 30 per module (minModuleSize = 30) and a threshold for merging similar modules set at 0.25 (cutHeight = 0.25). After network generation, preliminary predictions of the connections between transcription factors and their regulatory target genes were made. Network diagrams were visualized using Cytoscape 3.3.0 (Shannon et al. 2003).

### 2.6 Quantitative Real‐Time PCR Analysis

RNA was extracted from roots and stems, and its integrity was verified through 1% agarose gel electrophoresis. Quantitative primers were designed using Primer Premier 5 software, with 18s RNA serving as the internal reference. The QRT‐PCR reaction system comprised cDNA (2.0 μL, 100 ng μL^−1^), left primer (0.7 μL, 10 μmol L^−1^), right primer (0.7 μL, 10 μmol L^−1^), SYBR Green I Master Mix (Promega) (10.0 μL), and ddH_2_O (6.6 μL). Each sample underwent three biological replicates and two technical replicates to ensure reliable results.

### 2.7 Statistical Analysis

Basic statistical analyses were performed using DPS software (version 9.01) and GraphPad Prism 8.0.2. All experimental data are presented as mean ± standard deviation (SD), with each group having *n* = 3 samples. Differences between two groups were compared using Student's *t*‐test, and results are expressed as *p*‐values. A *p*‐value < 0.05 was considered statistically significant.

## 3 Result

### 3.1 The Influence of Arbuscular Mycorrhizal Fungi on Sugarcane Growth and Physicochemical Properties of Rhizosphere Soil

To investigate the effects of inoculating arbuscular mycorrhizal fungal inoculants on sugarcane growth and soil nutrient status, measurements were conducted on sugarcane plant height, stem diameter, chlorophyll content, root length, root surface area, brix, as well as soil pH, available nitrogen, soil organic matter, available phosphorus, total phosphorus, and available potassium.

**FIGURE 1:**
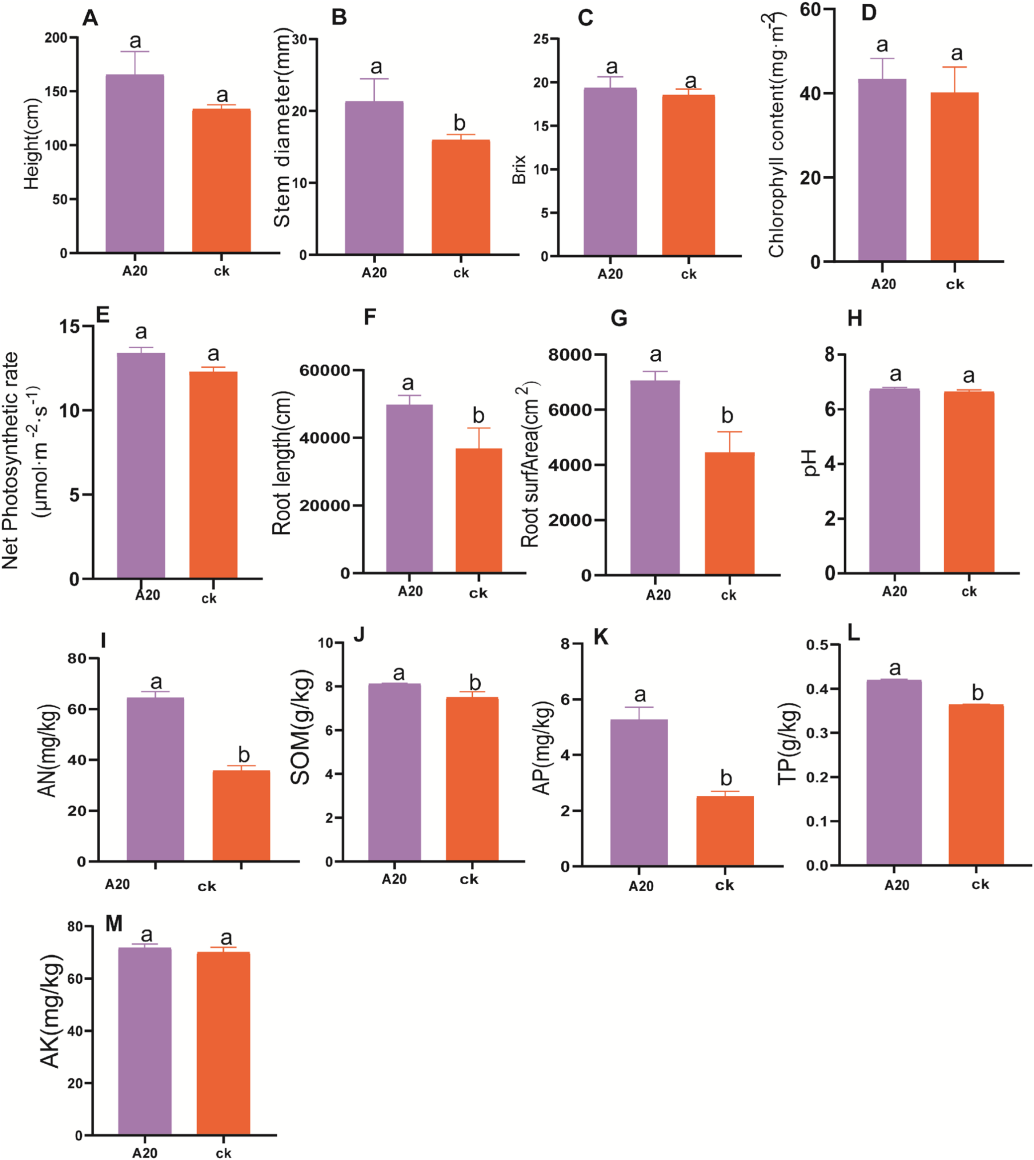
Analysis of the variations in sugarcane growth and root soil nutrient content under different treatment conditions. Different letters (a, b) represent significant differences (LSD, *p* < 0.05). (A) Plant height. (B) Stem diameter. (C) Brix. (D) Chlorophyll content. (E) Net photosynthetic rate. (F) Root length. (G) Root surfArea. (H) pH. (I) Available nitrogen (AN). (J) Soil organic matter. (K) Available phosphorus (AP). (L) Total phosphorus. (M) Available potassium (AK).

**FIGURE 2:**
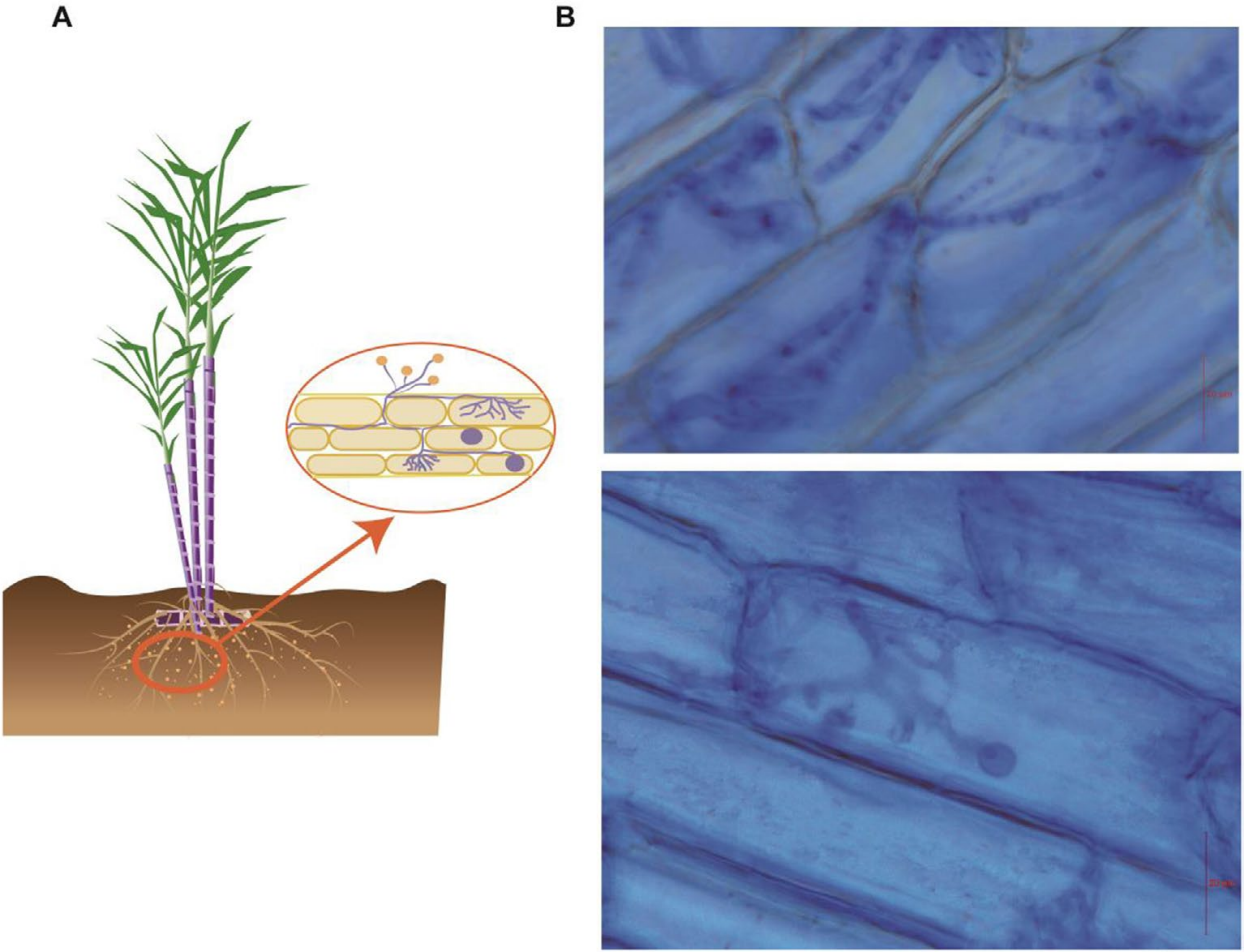
(A) Schematic diagram of arbuscular mycorrhizal fungi infecting sugarcane roots; (B) image of arbuscular mycorrhizal fungi infecting sugarcane roots.

Regarding sugarcane growth, inoculation with arbuscular mycorrhizal fungi significantly increased the stem diameter of sugarcane (*p* < 0.05) (Figure 1B). However, no significant differences were observed between the inoculated group and the control group in terms of plant height, brix level, chlorophyll content, and photosynthetic rate (Figure 1A,C–E). The presence of AM fungi demonstrably enhances subterranean sugarcane growth. Specifically, root length increased by 35.47% and root surface area by 58.57% in the presence of these fungi (*p* < 0.05), as depicted in Figure 1F,G. In regard to the colonization of AM fungi within sugarcane roots, we adopted the methodologies prescribed by BGC and Liao Nan et al. to calculate the colonization rate, which yielded an average value of 69.41%. This level of colonization is visually illustrated in Figure 2, showcasing the intricate network of hyphae that permeates the root system. Upon analyzing the physicochemical properties of the rhizosphere soil from pot‐cultivated sugarcane, it was found that the application of arbuscular mycorrhizal fungal inoculants significantly elevated the concentrations of available nitrogen, soil organic matter, total phosphorus, and available phosphorus in the soil (*p* < 0.05). Conversely, no significant impact was observed on soil pH or available potassium levels (Figure 1H–M).

### 3.2 RNA Sequence Analysis of Sugarcane Under Inoculation With Arbuscular Mycorrhizal Fungal Agents

To elucidate the key genes underlying sugarcane growth and nutrient uptake facilitated by arbuscular mycorrhizal fungi, transcriptome sequencing was conducted on the stems and roots of sugarcane during the elongation phase. Across the 12 libraries, a total of 250.48 million clean reads were generated, with minimum Q20 and Q30 quality scores of 96.02% and 92.82%, respectively, indicating high sequencing quality (Table S1). The alignment rates of these reads to the reference genome ranged from 85.11% to 87.49% (Table S2), suggesting a high degree of reliability in both sample collection and sequencing outcomes, thereby validating the suitability of the results for subsequent analyses.

**FIGURE 3:**
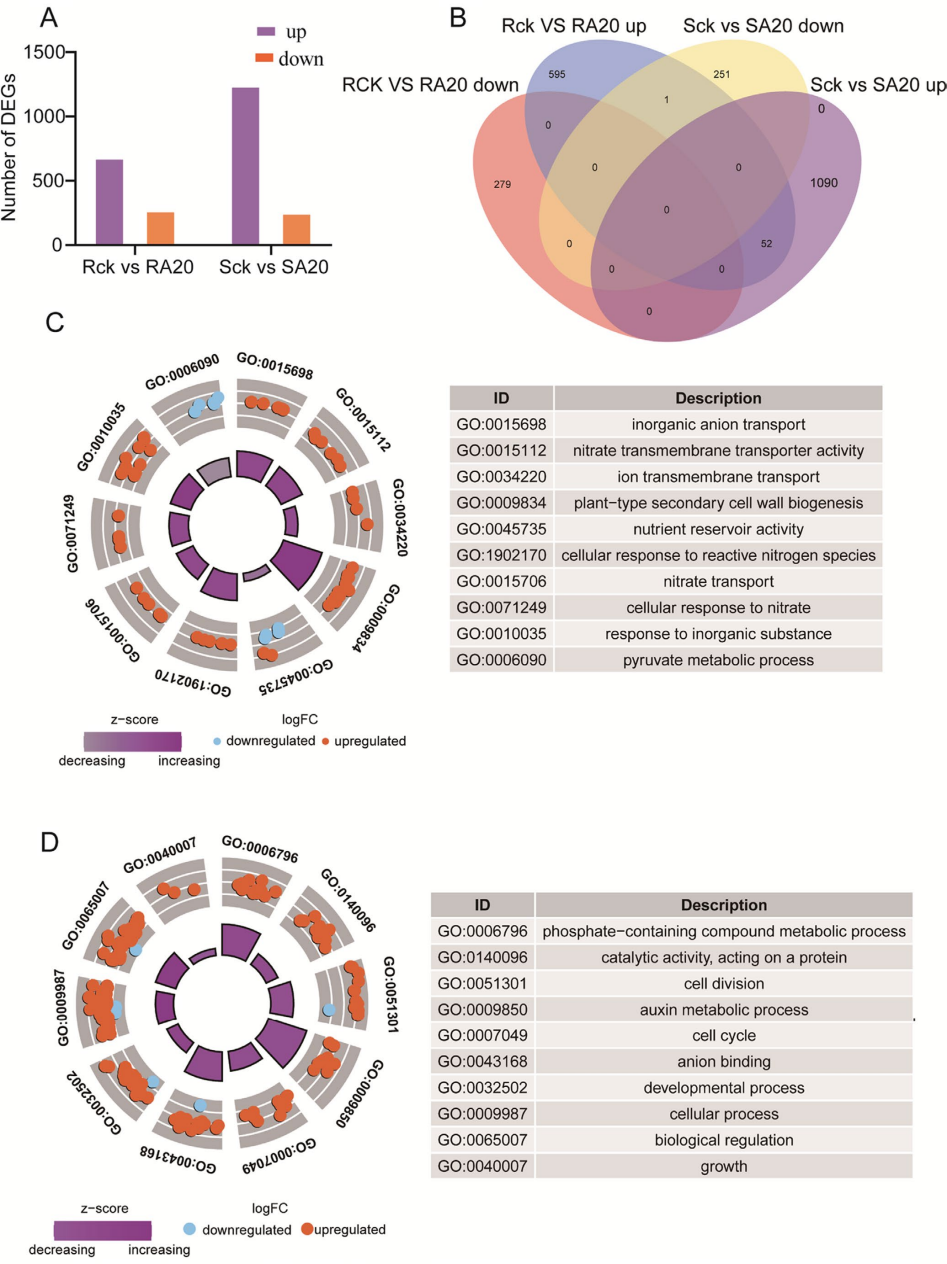
Difference analysis of gene expression by pairwise comparisons. Rck versus RA20: comparison of differentially expressed genes in roots between non‐inoculated and arbuscular mycorrhizal fungi‐inoculated plants; Sck versus SA20: comparison of differentially expressed genes in stems between non‐inoculated and inoculated arbuscular mycorrhizal fungi; (A) The number of DEGs in roots and stems after inoculation with arbuscular mycorrhizal fungi. (B) Venn diagram of DEGs in roots and stems under inoculation with arbuscular mycorrhizal fungi. (C) GO enrichment radar plot in Rck versus RA20. (D) GO enrichment radar plot in Sck versus SA20.

Differentially expressed transcripts (DEGs), rigorously interrogated according to stringent criteria of an absolute log2 fold change (|log2 FC|) threshold of ≥ 1 and a false discovery rate (FDR) threshold of ≤ 0.05, were comprehensively profiled in both roots and stems subsequent to arbuscular mycorrhizal fungal inoculation. Specifically, a comparative transcriptomic analysis between AM‐inoculated roots (RA20) and untreated control roots (Rck) discerned 927 DEGs, whereas the analogous comparison between AM‐inoculated stems (SA20) and untreated control stems (Sck) revealed 1394 DEGs. Within the root DEG cohort, 648 transcripts were designated as upregulated and 279 as downregulated. Conversely, in the stem DEG cohort, 1142 transcripts were upregulated and 252 were downregulated (Figure 3A). Notably, subsequent to AM fungal colonization, 59 DEGs consistently exhibited upregulation in both roots and stems, whereas a solitary DEG manifested an inverse expression pattern across the two tissues. Furthermore, a distinct subset of 874 DEGs was exclusively identified in roots, and an additional subset of 1341 was uniquely present in stems (Figure 3B).

The GO functional annotation analysis systematically categorized the DEGs into biological processes, cellular components, and molecular functions, providing insights into tissue‐specific responses following AM fungal inoculation. In roots, second‐level GO annotations highlighted processes associated with growth and nutrient acquisition, including “nitrate transport,” “inorganic anion transport,” and “plant‐type secondary cell wall biogenesis” (biological processes), as well as “nitrate transmembrane transporter activity” (molecular function). These findings suggest an enhancement of nutrient uptake mechanisms in roots, particularly concerning nitrogen assimilation (Figure 3C). Conversely, in stems, GO annotations predominantly encompassed biological processes such as “cell division,” “biological regulation,” and “growth,” along with molecular functions like “anion binding,” indicating that AM inoculation may facilitate growth and developmental processes in stem tissues (Figure 3D). Collectively, the results provide molecular evidence that AM fungal inoculation promotes sugarcane growth by enhancing nutrient transport and tissue development, thereby underscoring the positive influence of mycorrhizal symbiosis on crop productivity.

**FIGURE 4:**
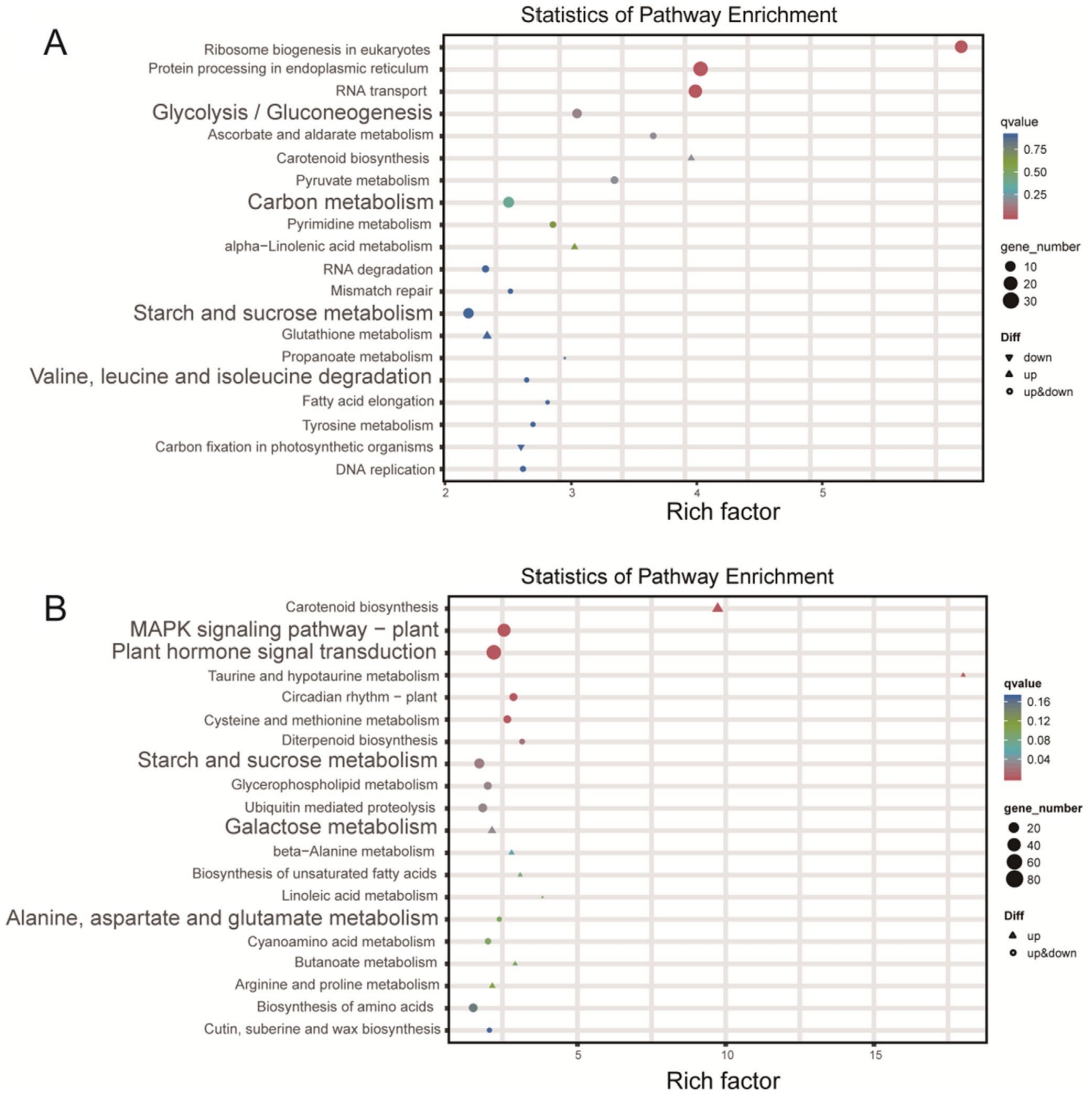
(A) KEGG pathway analysis of DEGs in Rck versus RA20. (B) KEGG pathway analysis of DEGs in Sck versus SA20.

To elucidate the functional categories and metabolic pathways associated with response genes in sugarcane treated with arbuscular mycorrhizal (AM) fungal inoculants, we performed pathway enrichment analysis using the KEGG database. In roots, a total of 93 significantly enriched pathways were identified, predominantly involved in carbohydrate, amino acid, and energy metabolism. Notable pathways included Glycolysis/Gluconeogenesis, Starch and Sucrose Metabolism, and Carbon Fixation, indicating enhanced metabolic activity related to energy generation and carbohydrate processing in root tissues (Figure 4A). In stems, 92 pathways were enriched, with a focus on signal transduction, carbohydrate, and amino acid metabolism. Key pathways such as MAPK Signaling, Plant Hormone Signal Transduction, and Galactose Metabolism were specifically highlighted, suggesting that AM inoculation influences both metabolic functions and signaling mechanisms associated with growth and developmental processes (Figure 4B). Collectively, these results demonstrate that AM fungal inoculants exert a profound impact on sugarcane's carbon and sugar metabolism, as well as its signal transduction pathways, which likely contribute to the observed growth promotion effects.

### 3.3 Identification of Key Transcriptional Regulators in Sugarcane Roots and Stems Following AM Fungal Inoculation via WGCNA

A rigorous weighted gene co‐expression network analysis was undertaken, focusing on integrating rhizosphere soil nutrient indices (AN, SOC, AP) as phenotypic data with root gene expression, and correlating stem‐specific gene expression with growth indices like plant height in sugarcane. Genes were carefully selected based on stringent criteria: an absolute Pearson correlation coefficient (*r*) exceeding 0.7 and a statistically significant *p*‐value less than 0.05 for initial filtering, followed by identifying core genes within modules using a threshold of k‐means module eigengene (KME) connectivity greater than 0.7.

**FIGURE 5:**
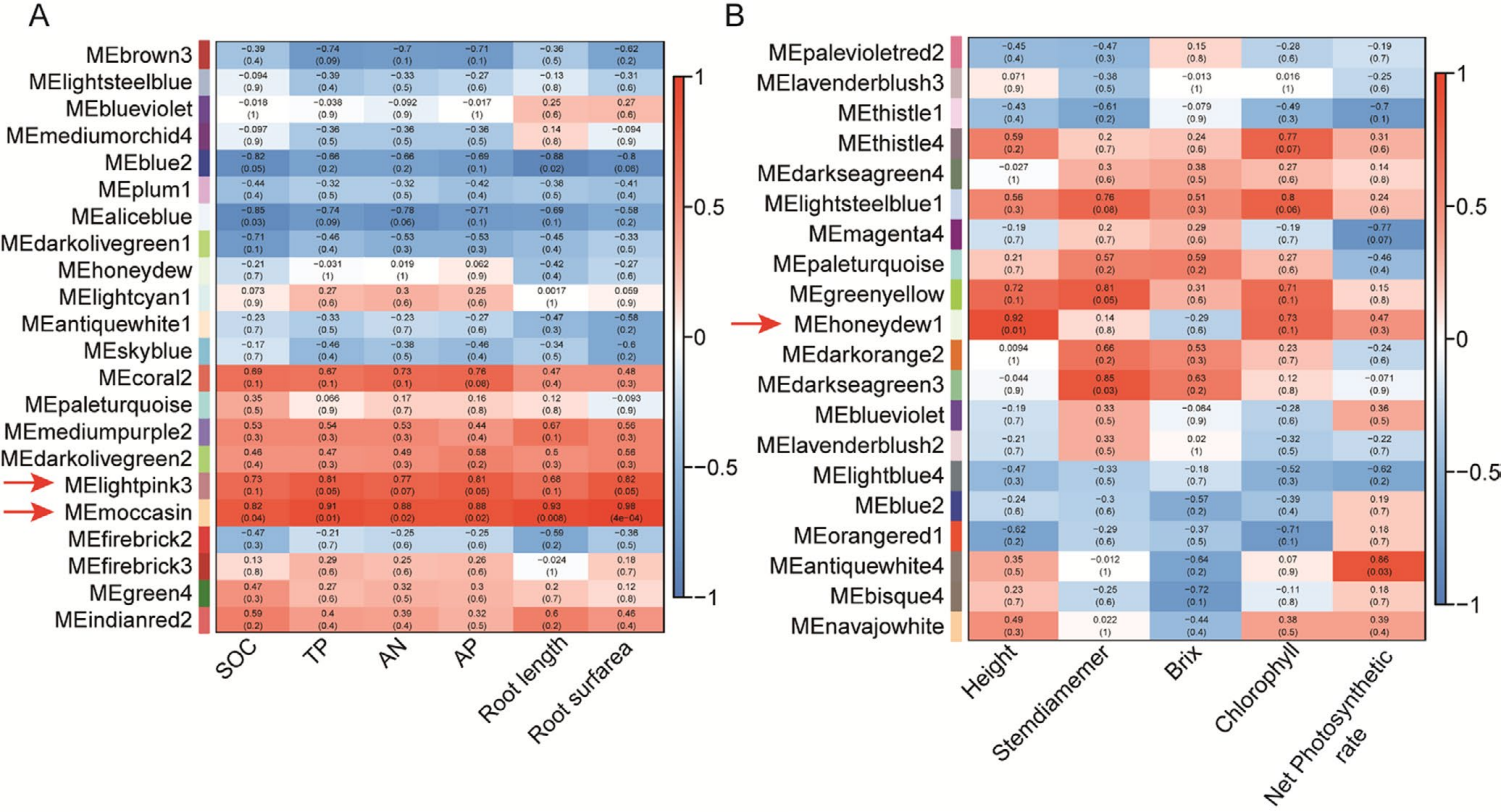
WGCNA module identification and correlation analysis. (A) Correlation between identified modules and root system growth and rhizosphere soil nutrient status following arbuscular mycorrhizal fungi inoculation. (B) Correlation between the identified modules and the physiological traits of sugarcane after inoculating with arbuscular mycorrhizal fungi. Red and blue color notes positive and negative correlation with gene expression, respectively.

As shown in Figure 5A, we have accurately identified two modules that exhibit statistically significant (*r* > |0.7|) correlations with nutrient uptake in root systems. Following the completion of the WGCNA, we conducted a thorough examination of the co‐expression networks within the MEmoccasin and MElightpink3 modules. Upon application of arbuscular mycorrhizal fungal inoculants, the MEmoccasin module demonstrated robustly positive correlations with rhizosphere soil organic carbon (*r* = 0.82, *p* = 0.04), total phosphorus (*r* = 0.91, *p* = 0.01), available nitrogen (*r* = 0.88, *p* = 0.02), and available phosphorus content (*r* = 0.88, *p* = 0.02). Additionally, it showed positive associations with root length (*r* = 0.93, *p* = 0.008) and root surface area (*r* = 0.98, *p* = 4e‐04). Similarly, the MElightpink3 module exhibited positive correlations with TP (*r* = 0.81, *p* = 0.05) and AP content (*r* = 0.81, *p* = 0.05), and also displayed notable correlations with other soil nutrient concentrations.

In this study, we observed a significant positive correlation between the MEmoccasin module and the nutrient content in the rhizosphere soil of sugarcane (Figure 4A). The associated network diagram of the MEmoccasin module is illustrated in Figure 4B, which facilitates the identification of key genes related to nutrient acquisition. Among the candidate hub genes identified within this module are *SoffiXsponR570.05Ag174900.v2.1* (Cellulose synthase A catalytic subunit 9, *CESA9*), *SoffiXsponR570.07Cg222700.v2.1* (Anthocyanidin reductase ((2S)‐flavan‐3‐ol‐forming), *ANR*), and *SoffiXsponR570.03Ag024900.v2.1* (Cyclin‐P4‐1, *CYCP4*). These genes are likely to play pivotal roles in the function of the MEmoccasin module. Specifically, *CESA9* is associated with cellulose synthesis, which may influence the structural integrity and functional capacity of the root system, thereby enhancing the sugarcane plant's ability to absorb soil nutrients. *ANR* is closely linked to the biosynthesis of flavonoids, which are critical for plant resilience and growth regulation. Meanwhile, *CYCP*4 is implicated in the regulation of the cell cycle, potentially affecting the growth rate and branching patterns of the root system. Furthermore, the KEGG pathway enrichment analysis revealed that the MEmoccasin module is significantly enriched in metabolic pathways related to “Flavone and flavonol biosynthesis” and “Flavonoid biosynthesis.” These pathways are not only crucial for plant growth and development but also play significant roles in the plant's response mechanisms to environmental stressors. Flavonoids are known to contribute to antioxidant defense, pest resistance, and the enhancement of soil microbial community structure, which may facilitate the effective utilization of nutrients. In summary, the investigation of the MEmoccasin module elucidates its critical role in the dynamics of nutrient availability in the rhizosphere of sugarcane. The functional implications of the identified hub genes and their associated metabolic pathways may provide new molecular targets for improving nutrient use efficiency and stress resilience in sugarcane. This finding not only offers potential avenues for genetic enhancement of sugarcane but also provides a theoretical basis for optimizing soil management strategies.

**FIGURE 6:**
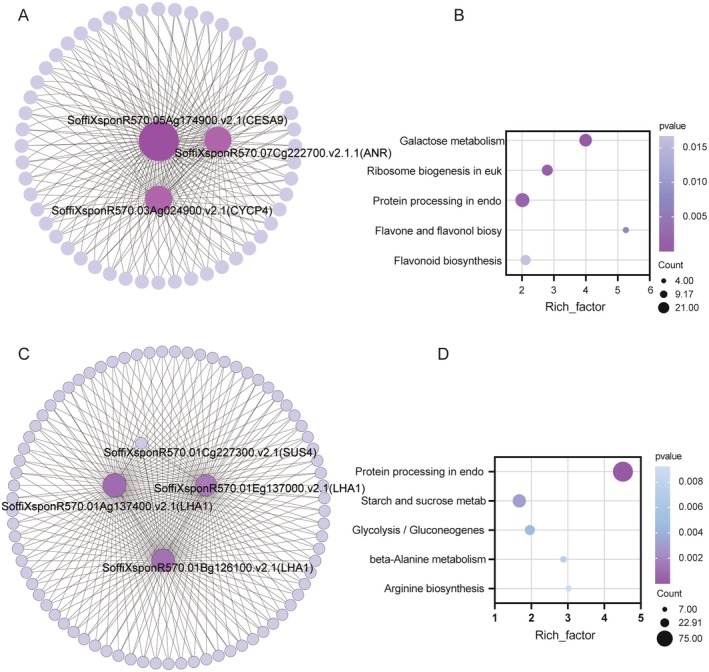
(A) Gene interaction network analysis in the MEmoccasin modules, the bigger the node, the greater the number of connections it has. The center area of the circle were candidate hub genes. (B) KEGG pathway enrichment analysis of the MEmoccasin modules. (C) Gene interaction network analysis in the MElightpink3 modules. (D) KEGG pathway enrichment analysis of the MElightpink3 modules.

The connectivity network visualization of the MElightpink3 module is exhibited in Figure 6C, wherein *SoffiXsponR570.01Ag137400.v2.1* (Plasma membrane ATPase 1, *LHA1*), *SoffiXsponR570.01Eg137000.v2.1* (*LHA1*), *SoffiXsponR570.01Cg227300.v2.1* (*SUS4*), and *SoffiXsponR570.01Bg126100.v2.1* (*LHA1*) have been computationally determined as candidate nodal genes. Notably, the preponderance of *LHA1* annotations underscores the potential significance of the *LHA1* regulatory cascade in modulating the influence of arbuscular mycorrhizal fungi on sugarcane root architecture and phosphorus uptake. Within the MElightpink3 module, three metabolic pathways have been identified as statistically enriched through rigorous bioinformatics analysis: “Protein processing in the endoplasmic reticulum,” “Starch and sucrose metabolism,” and “Glycolysis/Gluconeogenesis” (Figure 6D).

**FIGURE 7:**
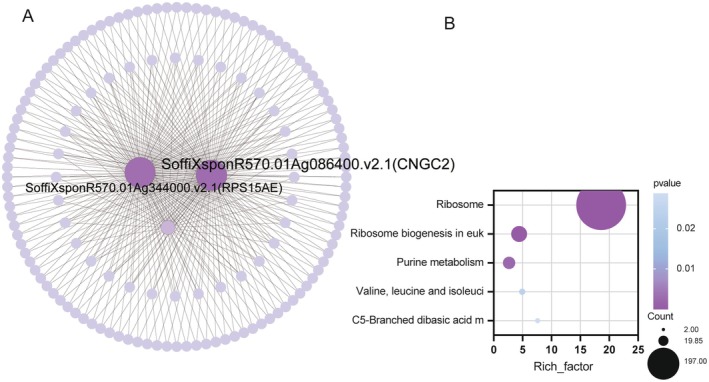
(A) Gene interaction network analysis in the MEhoneydew1 modules. (B) KEGG pathway enrichment analysis of the MEhoneydew1 modules.

As shown in Figure 5B, we systematically partitioned 22 modules that are closely related to sugarcane stem growth dynamics. In particular, the MEhoneydew1 module shows a statistically significant positive correlation with sugarcane plant height (*r* = 0.92, *p* = 0.01), while the MEantiquewhite4 module shows a strong positive association with leaf photosynthetic efficiency (*r* = 0.86, *p* = 0.03). Within the boundaries of the MEhoneydew1 module, an intricate correlation network was constructed, revealing *SoffiXsponR570.01Ag344000.v2.1* (40S ribosomal protein S15a‐5, *RPS15AE*) and *SoffiXsponR570.01Ag086400.v2.1* (Cyclic nucleotide‐gated ion channel 2, *CNGC2*) as the key genes that play central roles in regulating the functionality of the module (Figure 7A). Furthermore, KEGG pathway analysis revealed a prominent enrichment of these genes in the “Ribosome” and ‘Ribosome biogenesis in eukaryotes’ pathways (Figure 7B). Conversely, in the MEantiquewhite4 module, a thorough examination failed to identify any candidate genes with high connectivity that were also highly involved in core genetic processes related to sugarcane growth.

### 3.4 Validation of Gene Expression Changes by qRT‐PCR

**FIGURE 8:**
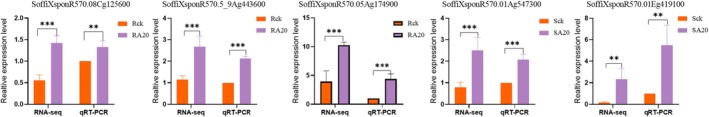
Real‐time quantitative PCR and RNA‐seq analysis of four genes.

To ensure the utmost precision and reproducibility in demonstrating the accuracy of our transcriptome profiling, we randomly selected a panel of four candidate genes and performed quantitative reverse transcription polymerase chain reaction (qRT‐PCR) analyses to precisely quantify their transcript abundances. The resulting qRT‐PCR results showed statistically significant and robust agreement with the expression patterns derived from RNA sequencing (RNA‐Seq). This meticulous validation process, which underscores the striking agreement between qRT‐PCR and RNA‐Seq data, clearly demonstrates the scientific rigor, precision, and reliability of our RNA‐Seq dataset (Figure 8).

## 4 Discussion

In the rigorous and integrated investigation of agricultural microbiology and plant physiology, the symbiotic association forged between AM fungi and the root system of host plants exerts profound and specific effects on the physiological metabolic regulation, growth dynamics, and developmental processes of the host plants, as well as the amelioration of soil physicochemical properties. This occurs via intricate biochemical and molecular mechanisms (Keunbae et al. 2022; Kong et al. 2018; Sun et al. 2022). Specifically, within the context of this study, during the elongation phase of sugarcane growth, AM fungal colonization significantly facilitated the augmentation of stem diameter. Furthermore, exhaustive research undertaken by scholars such as Kaldorf and Ludwig‐Müller in the maize system has elucidated the specific capacity of AM fungi to stimulate the biosynthesis of plant auxins, particularly indole‐3‐butyric acid (IBA) (Kaldorf and Ludwig‐Müller 2000). Similarly, studies in the tea plant system have precisely demonstrated the beneficial role of AM fungi in optimizing root morphological parameters, manifested as a substantial increase in total root volume, an intensification of lateral root density, and an elongation of lateral root length (Liu et al. 2024). These findings are congruent with the observations from the present study, which underscores the remarkable enhancement of sugarcane root length and root surface area by AM fungi, thereby reinforcing the academic rigor and precision of the conclusions presented.

In the exhaustive pursuit of agricultural sciences, elucidating the intricate interactions between soil characteristics and plant nutrient acquisition mechanisms represents a paramount task for securing sustainable crop production and preserving the vitality and stability of agricultural ecosystems. The present investigation conducts an in‐depth analysis of the impacts of AM fungal enrichment on soil nutrient profiles, unveiling several intriguing facets deserving of profounder scrutiny.

Foremost, the introduction of AM fungal inoculants significantly increased the SOM content within the sugarcane rhizosphere. On one hand, AM fungi facilitate SOM preservation by serving as long‐term carbon sinks, enhancing soil aggregate stability, and promoting the formation of mineral‐associated organic carbon (Li et al. 2024). On the other hand, AM fungi influence soil microbial communities, promoting the decomposition, re‐synthesis, and spatial reorganization of organic constituents, thereby enhancing the transformation and sequestration capacities of soil organic carbon (Frey 2019; Wu et al. 2024). This highlights the central role that AM fungi play in the terrestrial carbon cycle, where they regulate SOM generation, biogeochemical cycling, structural rearrangement, and stabilization processes (Wu et al. 2024). Moreover, the utilization of AM fungi displayed profound efficacy in augmenting soil phosphorus levels (both available phosphorus, and total phosphorus), a phenomenon that may be attributed to their ability to colonize plant roots and harness extensive hyphal networks that traverse the soil matrix, sequestering phosphorus resources and subsequently translocating them to the root cortex, thereby enhancing phosphorus accessibility and assimilation by sugarcane (Das et al. 2022). Lastly, AM fungal enrichment demonstrated a positive effect on soil ammonium nitrogen content, indicating their pivotal contribution to nitrogen cycling dynamics in soil. This finding suggests that AM fungi foster nitrogen transformation processes, intricately influencing biological nitrogen assimilation, the mineralization of organic nitrogen pools, biological nitrogen fixation, nitrification, and denitrification reactions, as well as nitrogen leaching phenomena, thereby optimizing nitrogen availability and utilization efficiency within the agroecosystem (Veresoglou et al. 2011).

Currently, we have yet to fully understand which specific genes are activated by AM fungi to promote sugarcane growth and nutrient uptake. Consequently, following transcriptome analysis, we employed WGCNA to identify gene modules and hub genes that are associated with sugarcane growth and nutrient uptake. This approach aligns with recent advancements in AM symbiosis research: for example, WGCNA was employed in bread wheat to identify co‐expressed modules containing NL genes and transcription factors, revealing potential regulatory mechanisms during AM symbiosis (Zhang, Zhong, et al. 2022). Our study extends the utility of WGCNA to sugarcane‐AM interactions, uncovering both conserved and crop‐specific regulatory networks.

Roots serve as essential organs for plants to acquire nutrients from soil and adapt to complex environmental conditions (Tracy et al. 2020; Zinta et al. 2022). Within the MEMoccasin module, we identified core genes linked to root growth and nutrient uptake, such as *SoffiXsponR570.05Ag174900.v2.1* (*CESA9*), *SoffiXsponR570.07Cg222700.v2.1* (*ANR1*), and *SoffiXsponR570.03Ag024900.v2.1* (*CYCP4*). While genes like *CESA9*, *ANR1*, and *CYCP4* have been well characterized in model plants and some crops, their roles in sugarcane, particularly in the context of AM fungi interactions, remain largely unexplored. Notably, previous transcriptomic studies in other crops, such as rice and Arabidopsis, have identified modules and hub genes associated with mycorrhizal symbiosis, some of which overlap with our findings, such as genes involved in cell wall biosynthesis and nutrient sensing. For example, *CESA* genes are known to be critical for cell wall integrity, and mutations in rice (e.g., *OsCESA9*) cause impaired growth (Wang et al. 2012), while in Arabidopsis, *CESA* regulation influences hypocotyl elongation via nitric oxide signaling (Li et al. 2023). Similarly, *ANR1*'s role in nitrate signaling and lateral root development has been documented across plant species (Remans et al. 2006), and its involvement suggests conserved mechanisms in nutrient response pathways (Zhang and Forde 1998). *PtrANR1* promotes drought tolerance by enhancing root development, augmenting proline accumulation, and scavenging reactive oxygen species (ROS) (Wan et al. 2023). Phosphorus is an indispensable macronutrient for plant growth and development. *OsCYCP4*, a member of the *CYCP* gene family, is involved in maintaining phosphorus homeostasis and signaling in rice (Xu et al. 2020). In the context of the MEMoccasin module, the enrichment of “Flavone and flavonol biosynthesis” pathways in our module aligns with prior studies demonstrating the role of flavonoids in mycorrhizal interactions. For instance, Tian et al. reported that flavonoid exudates can promote AM fungal colonization and symbiosis establishment, a mechanism that has been observed across diverse crops, including maize and soybean (Tian et al. 2021). In crops like maize, flavonoids have been shown to modulate rhizosphere microbiome composition, enhancing nutrient acquisition (Yu et al. 2021). Our findings thus extend these insights to sugarcane, suggesting that AM fungi may influence flavonoid biosynthesis to facilitate symbiosis and improve nutrient uptake.

In the MElightpink3 module, we have precisely identified *SoffiXsponR570.01Ag137400.v2.1* (*LHA1*), *SoffiXsponR570.01Eg137000.v2.1* (*LHA1*), *SoffiXsponR570.01Cg227300.v2.1* (*SUS4*), and *SoffiXsponR570.01Bg126100.v2.1* (*LHA1*) as the candidate hub genes. Arbuscular mycorrhizal fungi facilitate the translocation of solutes, including phosphates, to plant hosts in exchange for photosynthetic assimilates. Within this intricate process, the proton gradient generated by the proton H(+)‐ATPase serves as a pivotal mechanism driving solute uptake (Rosewarne et al. 2007). Notably, the H^+^‐ATPase subtype encoded by the *LHA1* gene is directly implicated in the establishment of the proton gradient necessary for phosphate uptake by epidermal cells (Ferrol et al. 2002), underscoring its central importance in symbiotic nutrient exchange. The role of *LHA1* as a proton pump in establishing proton gradients essential for nutrient uptake has been well documented in roots of various species. However, its specific association with AM fungi‐mediated nutrient exchange in sugarcane represents a novel insight. Furthermore, *SUS4*, a constituent of the Sucrose Synthase (*SUS*) family, displays bidirectional enzymatic activity that preferentially promotes sucrose synthesis in roots. Specifically, *SUS* catalyzes the conversion of fructose and uridine diphosphate glucose (UDP‐Glc) to sucrose and uridine diphosphate (UDP), a biochemical reaction that lies at the heart of sucrose metabolism and carbon allocation strategies in plants. The expression patterns of *SUS* exhibit distinct spatio‐temporal specificity, with various members, such as *OsSUS1* to *OsSUS4* in rice, displaying specific distributions across roots, leaves, and panicles (Hirose et al. 2008). Our study reveals that *SUS4* in sugarcane is predominantly expressed in roots and stems, highlighting its diverse functional roles in plant growth and development. These previous studies on model plants and other crops have provided a general understanding of the functions of *SUS* genes (Bieniawska et al. 2007). However, our study in sugarcane integrates *SUS4* within a specific gene module related to AM symbiosis, which was not comprehensively addressed in previous research. This integration offers a more detailed view of how *SUS4* participates in nutrient and energy metabolism in the context of AM—plant interactions. Given the significant enrichment of critical metabolic pathways such as “Starch and Sucrose Metabolism” and “Glycolysis/Gluconeogenesis” revealed by KEGG enrichment analysis in the MElightpink3 module, we posit that the gene network within this module, particularly the candidate hub genes including *LHA1* and *SUS4*, facilitates mineral nutrient absorption (e.g., phosphates), regulates the metabolic balance between sucrose and starch, and modulates glycolysis and gluconeogenesis pathways through proton pumping functions, carbon allocation, and nutrient exchange. Consequently, these genes play pivotal roles in nutrient absorption and energy distribution in plant roots.

The sugarcane stalk, being the fundamental organ for sugarcane ontogeny and proliferation, experiences processes such as growth velocity, dry matter accrual, and sugar accumulation, which directly correlate with the crop's yield and quality. Within the MEhoneydew1 module, we have pinpointed *SoffiXsponR570.01Ag344000.v2.1* (*RPS15AE*) and *SoffiXsponR570.01Ag086400.v2.1* (*CNGC2*) as pivotal genes implicated in plant growth dynamics. In the model plant Arabidopsis, *RPS15AE* operates as a modulator of the translational efficiency of ribosomal protein S15a, thereby influencing leaf, root, and cellular development. Conversely, *CNGCs*, a family of non‐selective cation channels gated by cyclic nucleotides, exhibit pivotal functions in mediating the transmembrane flux of essential cations like Ca^2+^, K^+^, and Na^+^, as well as in transducing signals in response to environmental stresses (Liu et al. 2018). Prior investigations have underscored the critical roles played by *CNGC* family members in regulating plant growth and development, as well as adapting to environmental fluctuations (Jarratt‐Barnham et al. 2021). Notably, *CNGC2* in Arabidopsis specifically acts to dampen disease resistance and thermotolerance while fostering plant growth (Lu et al. 2022). While the involvement of *CNGC*s in plant growth and stress response is well‐documented, their specific role in AM fungal interactions, particularly in sugarcane, remains largely unexplored. Previous transcriptomic studies on AM fungal interactions in other crops, such as cotton (Zhao et al. 2022) and rice (Wang et al. 2023), have identified changes in ion transport genes, but the specific *CNGC2* homolog identified here as a hub gene in sugarcane's response to AM fungi has not been prominently featured. This suggests a potentially unique role for this particular *CNGC2* isoform in mediating the AM symbiosis in sugarcane. It is noteworthy that our analysis revealed a significant enrichment of ribosome‐associated functions within the MEhoneydew1 module, underscoring the centrality of ribosomal activity in regulating gene expression patterns within this module. Ribosomes, the core machineries for protein biosynthesis, must function efficiently to ensure the timely synthesis of various regulatory molecules within cells. Based on these findings, we hypothesize that arbuscular mycorrhizal fungi may exert their influence on host plants by modulating the expression and functionality of ribosomal proteins, thereby regulating metabolic pathways and ultimately enhancing plant growth and development through intricate mechanisms. This modulation, particularly involving *RPS15AE* and *CNGC2*, may represent a sugarcane‐specific adaptation to the AM symbiosis, warranting further investigation to elucidate the precise molecular mechanisms involved and to determine its potential for improving sugarcane yield and quality. Interestingly, the MEantiquewhite4 module did not yield any genes that met our criteria for hub genes. This observation could be attributed to several factors. First, the MEantiquewhite4 module may be smaller and less densely connected compared to other modules, resulting in lower overall intramodular connectivity and a lack of nodes with sufficiently high connectivity to be considered hubs. Second, it is also possible that the absence of identified hub genes is due to limitations in the data, such as incomplete gene annotation or insufficient statistical power to detect subtle regulatory relationships. Further investigation is needed to fully understand the regulatory architecture of the MEantiquewhite4 module and to determine whether alternative hub gene identification methods or additional data could reveal key regulatory nodes within this module.

## 5 Conclusion

The aim of this study was to elucidate the effects and mechanisms of arbuscular mycorrhizal fungi on the growth and nutrient uptake of elongating ROC22 sugarcane. To this end, the transcriptional changes in sugarcane roots and stems before and after treatment were investigated, coupled with WGCNA. The results showed that AMF inoculation promoted the growth of both above‐ and belowground parts of sugarcane and significantly enhanced the accumulation of nutrients such as available nitrogen, soil organic matter, total phosphorus, and available phosphorus in the rhizosphere soil. In addition, WGCNA identified two specific modules related to root nutrient uptake and two specific modules related to aboveground growth of sugarcane. Of particular note, *CESA9*, *ANR*, *CYCP4*, *LHA1*, *SUS4*, *RPS15AE*, and *CNGC2* were identified as potential hub genes, each potentially playing a critical role in sugarcane growth and soil nutrient uptake.

## Author Contributions

**Lifang Mo:** visualization (equal), writing – original draft (equal). **Ziqin Pang:** supervision (equal), methodology (equal) . **Zhuowei Tan:** supervision (equal). **Qiang Liu:** methodology (equal), supervision (equal). **Yixian Jia:** supervision (equal). **Yijie Xiao:** supervision (equal). **Zhaonian Yuan:** conceptualization (equal), data curation (equal), project administration (equal), supervision (equal).

## Conflicts of Interest

The authors declare no conflicts of interest.

## Supporting information


Tables S1–S2.

## Data Availability

The datasets presented in this study can be found in the China National Center for Bioinformation (PRJCA040896). The names of the repository/repositories and accession number(s) can be found in the article/Supporting Information.
