# Supplementary material for: Transcriptome and WGCNA Reveal the Key Genes of Arbuscular Mycorrhizal Fungi in Regulating Sugarcane Growth and Nutrient Absorption
Source: Food Sci Nutr. 2025 Jul 3;13(7):e70508. doi: 10.1002/fsn3.70508 (PMC12224041; doi:10.1002/fsn3.70508)
Supplement: Supplementary file 1 — Tables S1–S2. [file FSN3-13-e70508-s001.docx]

Supplementary Table 1 Quality Control Metrics for Transcriptome Sequencing Data of Samples from Different Treatments

| Sample | ReadSum | BaseSum | GC(%) | N(%) | Q20(%) | Q30(%) |
| --- | --- | --- | --- | --- | --- | --- |
| RA201 | 21085251 | 6315461002 | 53.38 | 0.01 | 97.05 | 94.61 |
| RA202 | 19963027 | 5977275077 | 53.25 | 0.01 | 96.97 | 94.47 |
| RA203 | 19686887 | 5894673458 | 52.65 | 0.01 | 96.51 | 93.65 |
| Rck1 | 20164639 | 6035057401 | 51.71 | 0.01 | 96.96 | 94.44 |
| Rck2 | 20195931 | 6047168198 | 52.33 | 0.01 | 96.73 | 94.04 |
| Rck3 | 23516701 | 7043498033 | 51.73 | 0.01 | 96.15 | 92.93 |
| SA201 | 21172960 | 6341294945 | 53.51 | 0.01 | 97.19 | 94.88 |
| SA202 | 22520527 | 6741029530 | 53.67 | 0 | 97.36 | 95.15 |
| SA203 | 20265569 | 6070888337 | 53.29 | 0.01 | 96.9 | 94.34 |
| SAck1 | 21670810 | 6490066620 | 52.02 | 0.01 | 96.02 | 92.82 |
| SAck2 | 20176010 | 6040432422 | 52.57 | 0.01 | 96.86 | 94.25 |
| SAck3 | 20067585 | 6010140643 | 52.99 | 0.01 | 96.44 | 93.45 |

Supplementary Table 2 Statistics of sequence comparison results between sequencing data and reference genome

| Sample | Total Reads | Mapped Reads | Uniq Mapped Reads | Multiple Map Reads | Reads Map to '+' | Reads Map to '-' |
| --- | --- | --- | --- | --- | --- | --- |
| RA201 | 42,170,502 | 36,715,171 (87.06%) | 23,184,153 (54.98%) | 13,531,018 (32.09%) | 29,967,186 (71.06%) | 29,870,427 (70.83%) |
| RA202 | 39,926,054 | 34,467,289 (86.33%) | 21,934,505 (54.94%) | 12,532,784 (31.39%) | 27,944,496 (69.99%) | 27,851,690 (69.76%) |
| RA203 | 39,373,774 | 34,143,004 (86.72%) | 21,799,931 (55.37%) | 12,343,073 (31.35%) | 27,619,963 (70.15%) | 27,525,278 (69.91%) |
| Rck1 | 40,329,278 | 34,765,256 (86.20%) | 21,888,463 (54.27%) | 12,876,793 (31.93%) | 28,498,402 (70.66%) | 28,379,116 (70.37%) |
| Rck2 | 40,391,862 | 34,913,959 (86.44%) | 21,979,919 (54.42%) | 12,934,040 (32.02%) | 28,653,899 (70.94%) | 28,550,105 (70.68%) |
| Rck3 | 47,033,402 | 40,028,096 (85.11%) | 25,460,285 (54.13%) | 14,567,811 (30.97%) | 32,505,518 (69.11%) | 32,422,676 (68.94%) |
| SA201 | 42,345,920 | 36,963,280 (87.29%) | 23,213,304 (54.82%) | 13,749,976 (32.47%) | 30,466,921 (71.95%) | 30,349,106 (71.67%) |
| SA202 | 45,041,054 | 39,404,739 (87.49%) | 24,547,695 (54.50%) | 14,857,044 (32.99%) | 32,695,978 (72.59%) | 32,527,979 (72.22%) |
| SA203 | 40,531,138 | 35,237,650 (86.94%) | 22,523,834 (55.57%) | 12,713,816 (31.37%) | 28,583,311 (70.52%) | 28,460,378 (70.22%) |
| SAck1 | 43,341,620 | 37,506,963 (86.54%) | 23,459,793 (54.13%) | 14,047,170 (32.41%) | 30,927,344 (71.36%) | 30,862,419 (71.21%) |
| SAck2 | 40,352,020 | 35,078,562 (86.93%) | 22,296,213 (55.25%) | 12,782,349 (31.68%) | 28,546,056 (70.74%) | 28,438,928 (70.48%) |
| SAck3 | 40,135,170 | 34,910,416 (86.98%) | 22,024,903 (54.88%) | 12,885,513 (32.11%) | 28,559,820 (71.16%) | 28,481,296 (70.96%) |
